# Dynamic interactions of physiological systems during competitive gaming: insights from network physiology - case report

**DOI:** 10.3389/fnetp.2024.1438073

**Published:** 2024-09-11

**Authors:** Andreas Stamatis, Grant B. Morgan, Jorge C. Reyes

**Affiliations:** ^1^ Health and Sport Sciences, University of Louisville, Louisville, KY, United States; ^2^ Sports Medicine Institute, University of Louisville Health, Louisville, KY, United States; ^3^ Educational Psychology, Baylor University, Waco, TX, United States

**Keywords:** heart rate, skin temperature, pupil dilation, Starcraft, temporal lag, time delay stability, ADHD

## Abstract

This study investigates the dynamic interactions between physiological systems during competitive gaming, utilizing a Network Physiology approach. By examining the physiological responses of a gamer with attention-deficit/hyperactivity disorder playing a real-time strategy game, we explore the relationships and temporal lag effects between pupil dilation, skin temperature, and heart rate. Our findings highlight the interconnectedness of these physiological systems and demonstrate how different physiological states are associated with unique patterns of network interactions. The study employs the concept of Time Delay Stability towards a deeper understanding of the complex dynamics involved. This research contributes to the growing field of Network Physiology by offering new insights into the physiological underpinnings of competitive gaming, potentially informing targeted training and recovery protocols for eSports athletes.

## 1 Introduction

The competitive landscape of *electronic sports* (eSports) has undergone a remarkable transformation over the past decade, evolving from a niche pastime into a mainstream entertainment and professional endeavor (Yong Jin, 2021). With millions of fans worldwide and significant financial investments, eSports tournaments now rival traditional sports in terms of viewership and revenue (Gawrysiak et al., 2020). Among the various genres within eSports, real-time strategy games, especially those set in open-world environments, stand out due to their demand for rapid decision-making, strategic planning, and precise motor coordination (Jordan and Dhamala, 2022). These games present an ideal setting for examining the physiological responses of players, given the potential for intense cognitive and emotional demands they require.

Attention-deficit/hyperactivity disorder (ADHD) is a behavioral condition characterized by difficulties in maintaining attention, organizing tasks, effective planning, and exercising caution before acting (APA, 2024). These challenges are particularly relevant in the context of eSports, where sustained attention and strategic planning are critical (Valls-Serrano et al., 2022). A recent review indicated that individuals with ADHD often exhibit atypical autonomic nervous system (ANS) functioning, especially hypo-arousal during resting states and tasks requiring sustained attention (Bellato et al., 2020). This ANS dysfunction may exacerbate the challenges faced by individuals with ADHD in high-demand environments like eSports, where optimal cognitive and emotional regulation is critical for success. While many studies suggest reduced ANS activity in ADHD, clear differences between individuals with and without ADHD have not been consistently found in the presence of stimulant medications. Stimulant medications have been shown to increase ANS activity and, in several cases, produce similar effects on ANS function (Idrees et al., 2023).

Physiological measures, such as pupil dilation, skin temperature, and heart rate are critical indicators of the body’s autonomic responses (e.g., Persiani et al., 2021; Schumann et al., 2020; Shehu et al., 2023). Pupil dilation, regulated by the autonomic nervous system, is a well-documented response to cognitive load and emotional arousal (Skaramagkas et al., 2023). During tasks that require significant mental effort and decision-making, such as gaming, pupils tend to dilate, reflecting heightened cognitive engagement and arousal levels. Previous studies have shown that pupil dilation can be used as a proxy for measuring cognitive workload and emotional intensity in various contexts, including gaming (Gorin et al., 2024). Concerning adults with ADHD, further research is required in the field of pupillometry due to the limited studies assessing that group (Bellato et al., 2020).

Similarly, skin temperature is another vital parameter that fluctuates in response to stress and cognitive workload. The thermoregulatory processes controlled by the sympathetic nervous system cause changes in skin temperature, with vasoconstriction leading to decreased peripheral temperature during periods of high stress (Cramer et al., 2022). Research has indicated that skin temperature can decrease during intense gaming sessions, reflecting the body’s physiological adaptation to sustained cognitive and emotional stress (Robinson et al., 2020). Reduced electrodermal activity (EDA) has been observed during resting-state periods in adults diagnosed with ADHD (e.g., Hermens et al., 2004).

Heart rate is a well-established measure of physiological arousal, and it may exhibit notable changes during gaming in concordance with gameplay sequences (e.g., Andre et al., 2020; Martin-Niedecken et al., 2021). During events with increased cognitive demand and/or emotional stress, one’s heart rate may concurrently or subsequently increase, which could signal the body’s preparation for action (Quadt et al., 2022). Furthermore, heart rate variability can provide insights into the autonomic nervous system’s balance between sympathetic and parasympathetic activity, which is crucial for understanding the physiological state of gamers during high-stake gameplay (Rodrigues et al., 2021). In terms of heart rate and related measures, there are no notable differences between adults with ADHD and those without ADHD, either during resting state or task-specific conditions (e.g., Lackschewitz et al., 2008; Oliver et al., 2012).

Although examining physiological responses to external stimuli in isolation of each other could offer valuable insights, a more comprehensive understanding of physiological response requires investigating the concordance and timing of the multiple systems’ interactions over time (Ivanov, 2021). Network Physiology focuses on the interconnectedness of physiological systems and emphasizes the need to study these interactions to grasp the holistic physiological state (Balagué et al., 2020). The Network Physiology approach is particularly relevant in eSports because rapid and dynamic gameplay can lead to concurrent and interacting physiological responses (Cranmer et al., 2021) and examining how physiological systems interact can provide useful insight into the complex dynamics of the autonomic nervous system during gaming.

The temporal lag effects between these physiological responses are also critical for understanding these interactions (e.g., Rizzo et al., 2020). Temporal lag refers to the delay between the onset of one physiological response and subsequent changes in another (Nijhawan, 2008). Lag effects can showcase the sequential nature of physiological adaptations during gameplay as an indication of the body’s integrated response mechanisms. This temporal dimension is essential for comprehensively understanding how different physiological systems influence each other over time during the gaming experience (Mortazavi et al., 2024).

A recent study in the field of eSports and Network Physiology examined the physiological responses of a player during NBA2K gameplay, marking the first instance of applying Network Physiology concepts to eSports (Stamatis et al., 2023). This study highlighted the interconnected nature of biological processes and laid the groundwork for further exploration. However, it primarily focused on two physiological systems, the brain and the eyes. The preliminary evidence of the brain’s and eyes’ network physiology reveals a significant gap in the literature regarding the comprehensive interactions and temporal dynamics of multiple physiological responses within the context of eSports.

There is a lack of research integrating the domains of ADHD, eSports, and Network Physiology. Therefore, to extend this line of research and address these gaps, this study aims to explore the following research questions:

1. How do the interactions between pupil dilation, skin temperature, and heart rate change during a real-time interspace-based strategy game?
2. How do these physiological responses (pupil dilation, skin temperature, and heart rate) interact over time, and what are the temporal lag effects between these organ systems during gaming?

By addressing these questions, this study seeks to contribute to the growing body of knowledge in Network Physiology within the context of eSports, providing a deeper understanding of the physiological dynamics that underpin competitive gaming experiences.

## 2 Methods

For this study, a participant was fitted with multiple devices to monitor various organ systems, such as eyes, skin, and heart, across 3 days in August-September 2022. During the 3 days of data collection, the participant engaged in multiple online activities, including web browsing and playing games of varying types and stimulation levels.

In more detail, on August 26th, the participant’s activities included a 3-min baseline, multiple sessions of Starcraft 2 (ranging from 2 to 14 min each), 4 min of DRG, and approximately 20 min of Overwatch, totaling approximately 91 min of recorded data. On September 2nd, the activities comprised a 3-min baseline, 17 min of Just Cause 4, 6 min of Vermintide 2, 12 min of Payday 2, two sessions of Starcraft 2 (9 and 8 min), two capsaicin EEG tests (2.5 min each), 20 min of ForTheKing, and 20 min of Imperator, totaling approximately 100 min of recorded data.

For the purposes of examining the relationships between organ systems, we used data from all sessions across the 2 days spent playing a real-time interspace-based strategy game from Blizzard Entertainment (i.e., Starcraft). Starcraft 2 was selected because it is among the most cognitively demanding games and requires simultaneous attention to multiple elements, including sequencing them properly. It creates a perception of being overwhelmed and this was considered a desirable effect to observe the participant’s responses. Activities were chosen to provide a range of cognitive and emotional stimuli, with Starcraft 2 offering complex gameplay and other games presenting contrasting demands. Capsaicin EEG tests were included to assess neural responses under controlled stress. Sessions were conducted under consistent lighting and environment (i.e., ∼72 F, ambient lighting during the daytime, minimal to no artificial lighting used), with no external stimulants used to ensure data consistency. Detailed logs were maintained for each day, recording the duration of each activity to ensure comprehensive assessment and analysis.

The participant was a 26-year-old Korean male, who is a gamer and neurotechnology enthusiast, active with many “neurohacking” communities and student clubs. The participant indicated that they have been diagnosed with ADHD. Stimulant medications were administered according to prescribed medical guidelines. The participant was free from cardiovascular disease (CVD), neural conditions, and had good vision.

### 2.1 Physiological measures/organ systems

Pupil dilation was utilized as an indicator of autonomic nervous system response. During the gaming sessions, the participant’s pupil diameter was continuously measured for both eyes using a Tobii^©^ wearable eye tracking device. The participant was seated in front of a 24″ monitor (1080p60 fps; same frequency and refresh rate in the recorded video captured), positioned approximately 15 inches from a Tobii 5L Eye Tracker (Tobii AB, Sweden). The monitor was connected to a gaming PC, and the video output was captured using an Elgato HD 60 S+ (Elgato Systems GmbH, Germany) capture card at a native frame rate of 60 fps.

**FIGURE 1:**
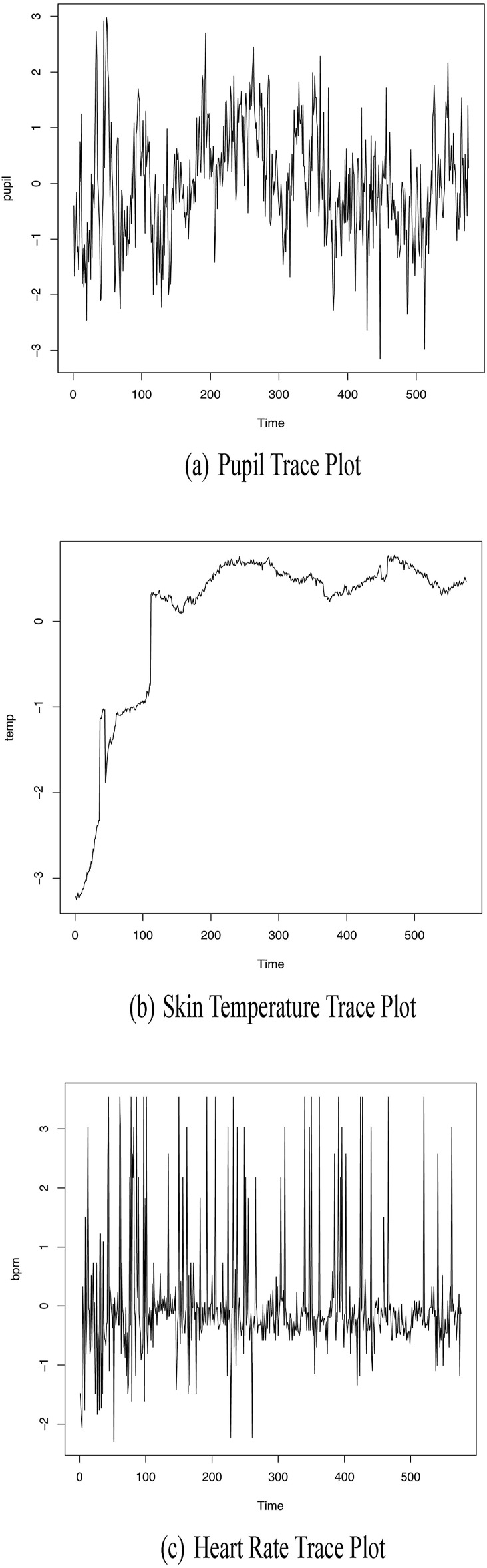
Plots of **(A)** pupil dilation, **(B)** skin temperature, and **(C)** heart rate series.

The pupil diameter data was collected using the Tobii 5L Eye Tracker, which employs corneal reflection techniques by illuminating the eye with infrared light and measuring the reflections from the cornea and pupil. The data was sampled at a rate of 120 Hz. A recent study evaluating the Tobii 5 Eye Tracker reported a median error of 30.70 pixels (0.65°) and an interquartile range of 33.37 pixels (0.71°), indicating the precision and consistency of the measurements (For further details on the accuracy and precision of the Tobii 5L Eye Tracker, refer to Housholder et al. (2022)). For the analytic model, the pupil dilation data was averaged across both eyes and segmented into four-second bins. These bins were then matched with other physiological data recorded during the gaming sessions. A total of 643 segments of matched data were obtained, corresponding to approximately 43 min of gameplay. The trace plot of the averaged pupil diameter data within each bin is depicted in Figure 1A.

Skin temperature was utilized as an indicator of thermoregulatory response and was continuously measured using an Emotibit^©^ (Connected Future Labs, United States) thermopile sensor, specifically the MLX90632 Thermopile embedded in the Emotibit board, operating at 7.5 hz in the Emotibit implementation, calibrated for the standard range of physiological temperatures (35°C–43°C). Emotibit is a device equipped with multiple biometric sensors designed to study nervous system activation. The physiological sensors on EmotiBit include electrodermal activity (EDA), photoplethysmography (PPG), skin conductance response amplitude (SCR amp), skin conductance response rise time (SCR ris), skin conductance response frequency (SCR freq), skin conductance response recovery time (SCR rec), interbeat interval (IBI), heart rate (HR), heart rate variability (HRV), blood oxygen saturation (SpO2), and respiration rate (RR). For further details on the validation of those sensors refer to Montgomery et al. (2024). The sensor was worn on the participant’s arm to ensure consistent skin contact, allowing for accurate heat transfer assessment. For the analytic model, skin temperature data was binned into four-second segments that were matched with other physiological data recorded during the gaming sessions. The trace plot of the averaged thermopile data within each segment is depicted in Figure 1B.

Heart rate, measured in beats per minute, was used as an indicator of cardiovascular response. The participant’s heart rate was continuously recorded using the photoplethysmogram (PPG) feature of the Emotibit device. The sensor was worn on the participant’s arm to ensure consistent skin contact, allowing for accurate heart rate assessment. Emotibit utilizes three LEDs for PPG analysis—red, infrared, and green—operating at a sample rate of 25 Hz. For the analytic model, heart rate data was binned into four-s segments and matched with other physiological data recorded during the gaming sessions. The trace plot of the averaged heart rate data within each bin is depicted in Figure 1C.

### 2.2 Preprocessing

The raw data was collected using the Lab Streaming Layer (LSL) stream and stored in the native XDF format. LSL provides excellent timestamp management, ensuring synchronization across data streams. Each physiological measure (pupil, EEG, and PPG) was synchronized to a common system clock and tracked in separate dataframes.

Following this, the data underwent basic de-noising, wherein data points exceeding four standard deviations (SD) from the dataset mean were removed. To further reduce noise in pupil and gaze measurements, we applied Pandas’ exponentially weighted smoothing (EWM) algorithm with a smoothing factor (alpha) of 0.7. This was done for both left and right pupil data to obtain smoothed signals. Next, we calculated the difference between consecutive smoothed values to capture changes over time. A rolling mean was then applied to these differences using a window size determined by multiplying the frame rate of the gaze data by a window duration of 0.33 s. This approach ensures that the resulting data is both smoothed and resistant to spiking, providing a cleaner signal for downstream analysis.

Both the raw and preprocessed data were then made available for a final processing pass, during which the recordings were segmented into four-second intervals. This segmentation technique allowed for the analysis of long recordings by showing trends over time while breaking the data into discrete “bins.” Each bin calculates data independently, thereby eliminating the need for resampling to match sample rates for comparison and analysis.

### 2.3 Analysis

The primary goal of the study was to examine the nature and strength of the relationship between the organ systems. As noted, we matched all three systems on the same four-second segments. The game play observations were collected across multiple sessions so prior to conducting the analyses, each stream of physiological data was normalized within session to have mean of zero and variance of one. Next, each stream of data was coded as time series. We then used the sample cross-correlation function (CCF) to not only examine the relationships between the physiological systems within segment but also whether one organ series might be related to past lags of another organ series. Finally, pupil dilation for any given segment was regressed onto lag retrospective times lags for pupil dilation as well as current and retrospective time lags for skin temperature and heart rate. All analyses were conducted using R with base commands and those from the astsa package (Stoffer and Poison, 2024). To ensure transparency and reproducibility of our findings, we have provided a sample script in the Supplementary Material.

## 3 Results

### 3.1 Pupil dilation and heart rate

The cross-correlation analysis revealed a weak, lagged correlation between pupil dilation and heart rate, with a peak correlation coefficient 0.1 at a lag of −12 s and −30 s. This suggests that increases in average pupil dilation in a given segment was followed by a slight change in heart rate at increments of 12 and 30 s later. The lag observed across participants indicates slight potential for integrated physiological response to a game-based stimulus by eyes and the heart albeit at differential rates.

### 3.2 Skin temperature and pupil dilation

The cross-correlation analysis revealed a weak but stronger lagged correlation between pupil dilation and heart rate, with a peak correlation coefficient 0.2 at a lag of −32 s. This suggests that increases in average pupil dilation in a given segment was followed by a thermoregulatory response 32 s later. The lag observed in the participant indicates a potential for integrated physiological response to a game-based stimulus by eyes and the skin temperature albeit at differential rates.

### 3.3 Lagged regression

In the lagged regression model, we initially regressed the three nearest pupil segments and 20 four-s lags of skin temperature and heart rate onto pupil dilation for a give segment. In the interest of model parsimony, we reduced the model to the effects that were most consistent with the descriptive output reported above. That is, the final model included the adjacent segment (i.e., 4-s lag) and two-segment lag (i.e., 8-s lag) for pupil dilation, the eight- and nine-segment lag for skin temperature (i.e., 32- and 36-s lag) and three-segment (i.e., 12-s lag) for heart rate. The model explained 43% of the variability in pupil dilation, although only pupil lags and skin temperature lags were statistically significant. Interestingly, the signs of the skin temperature lags were reversed, which suggests a reversal in the physiological interaction. That is, at a 32-s lag there is a positive relationship between pupil dilation and skin temperature, but the relationship reverses at 36-s lag. In other words, the skin temperature seems to respond on a 32-s delay from the pupil stimulation, but then drops at 36 s. The parameter estimates are provided in Table 1 above.

**TABLE 1:** Parameter estimates from lagged regression.

Parameter	Estimate	Standard error	*t* value	*p* value
Intercept	0.00	0.03	0.13	0.89
Pupil_Lag1	0.54	0.04	12.85	<0.001
Pupil_Lag2	0.15	0.04	3.53	<0.001
Temp_Lag8	0.84	0.39	2.17	0.03
Temp_Lag9	−0.82	0.38	−2.13	0.03
HR_Lag3	0.05	0.03	1.42	0.16

Model *R* ^2^ = 0.43, F_5,550_ = 81.31, *p* < 0.001.

**FIGURE 2:**
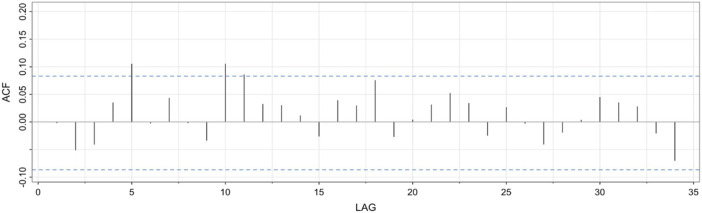
Residual autocorrelation function plot.

The autocorrelation function plot of the residuals showed minor residual autocorrelation at lag 5 and 10, but across the full series did not show significant remaining autocorrelation (See Figure 2). The plot indicates that the autocorrelation in the data has been, by and large, accounted for by the lag effects in the model.

## 4 Discussion

We draw from the field of Network Physiology to investigate the nature and strength of the relationship between pupil dilation, skin temperature, and heart rate during a real-time interspace-based strategy game and how these physiological responses interact over time (including the temporal lag effects between these organ systems during gaming). Our approach involved analyzing the dynamic interactions between three physiological systems to estimate how these networks contribute to overall physiological states and transitions between them.

Directly comparable studies have not been conducted in the context of eSports. Consequently, direct comparisons with previous findings are not feasible; however, our results align with previous evidence in the field of Network Physiology, particularly regarding the dynamic interactions and coupling between organ systems (e.g., Garcia-Retortillo and Ivanov, 2022). Prior studies have demonstrated that physiological systems exhibit complex, time-varying interactions that are essential for maintaining homeostasis and responding to internal and external stimuli (e.g., Balagué et al., 2022). In addition, the work by Balagué and Garcia-Retortillo (2022) highlighted the importance of *time delay stability* (TDS) method in Network Physiology when attempting to quantify the stability/consistency of the time delay of one system followed by another system. Lastly, our findings are consistent with previous work that a) supports the idea that pupil dilation is linked to autonomic responses, which in turn affects heart rate and b) indicates a direct relationship between cognitive load (pupil dilation) and subsequent sympathetic responses affecting skin temperature (Schumann et al., 2020).

From a methodological perspective, our study offers support for using CCF in eSports, despite its use in Network Physiology in other domains (e.g., Bashan et al., 2012). This and related-TDS methods have been used to study other complex physiological processes and conditions, enhancing our understanding of systemic regulation and coordination in this specific domain. From a practical perspective, understanding and cataloging biometric profiles of individuals can significantly enhance performance and recovery strategies in eSports. By analyzing how physiological responses such as heart rate, pupil dilation, and skin temperature vary under different gaming conditions, coaches can design personalized training programs. These programs can optimize the synchronization between physiological systems, leading to improved focus and faster recovery times. For example, if a player exhibits significant heart rate variability during high-stress moments, targeted interventions like biofeedback (e.g., neurofeedback; Arns et al., 2014) training can help regulate their autonomic responses, maintaining optimal performance under pressure. Similarly, awareness of (potential) ANS hypo-arousal in (non-medicated) individuals with ADHD can lead to tailored strategies that improve attention and strategic planning during gameplay. This personalized approach ensures that each player’s unique physiological needs are addressed, enhancing overall performance and well-being.

This study adds to the literature by providing an application of the novel Network Physiology concept to a rapidly growing and highly popular field. Whereas previous research has explored physiological networks during sleep and other states (e.g., Ivanov and Bartsch, 2014), our study examined time lag stability in the interactions between physiological systems in eSports. Specifically, we demonstrate that the TDS approach can reveal consistent time lags between bursts of activity in different systems, offering a more precise measure of the temporal coordination and stability of these interactions in eSports. This nuanced understanding of the dynamic interplay between physiological systems in eSports offers a new, preliminary perspective on how these networks are organized and restructured in response to different physiological states within in the eSports domain. Additionally, our use of a single-subject design, although limited in generalizability, allows for a more granular examination of intra-individual variability and the unique characteristics of network dynamics in response to specific states.

Like all studies, there are notable limitations to our study. One significant limitation is the collection, analysis, and interpretation of data from a single individual. This restricts the generalizability of our findings and does not capture the variability in physiological network dynamics across a broader gaming population. Additionally, the reliance on specific types of physiological data might overlook other relevant interactions involving different physiological systems.

Future research should focus on addressing these limitations by incorporating larger and heterogeneous gaming populations to allow examination of the variability and robustness of physiological network interactions. Moreover, expanding the scope of physiological data to include additional organ systems and employing advanced analytical methods can provide a more comprehensive view of the network dynamics. Longitudinal studies that track changes in these networks over time and in response to various interventions or pathological conditions could also offer valuable insights into the mechanisms underlying physiological (dis)regulation.

In conclusion, answering the call of Stamatis et al. (2023) for replication and addition of more systems/biological processes, our eSports study contributes to the growing body of literature in Network Physiology by providing preliminary evidence of distinct network dynamics associated with different physiological states and by highlighting the importance of time lag stability in understanding these interactions. We hope our findings serve as a catalyst for future and broader hypothesis-driven eSports research aimed at understanding the complex interactions between physiological systems within gaming environment and beyond. For instance, does the reversal in skin temperature responses reflect a homeostatic adjustment process after the initial regulatory mechanism reduces skin temperature? If so, could this be attributed to the presence of an ADHD diagnosis?

## Data Availability

The raw data supporting the conclusions of this article will be made available by the authors, without undue reservation.
