# Supplementary material for: Dynamic interactions of physiological systems during competitive gaming: insights from network physiology - case report
Source: Front Netw Physiol. 2024 Sep 11;4:1438073. doi: 10.3389/fnetp.2024.1438073 (PMC11422231; doi:10.3389/fnetp.2024.1438073)
Supplement: Supplementary file 1 [file Table1.DOCX]

CCF + Regression with Biometric Data

Andreas Stamatis, Grant B. Morgan & Jorge Carmona

2024-03-03

star <- read.csv("~/Alldata_Starcraft_grandcenter.csv", header=FALSE)

star <- star[complete.cases(star), ]

pupil<-star$V5

bpm<-star$V6

temp<-star$V7

**library**(astsa)

pupil<-ts(pupil)

bpm<-ts(bpm)

temp<-ts(temp)

plot(pupil)

plot(temp)

plot(bpm)


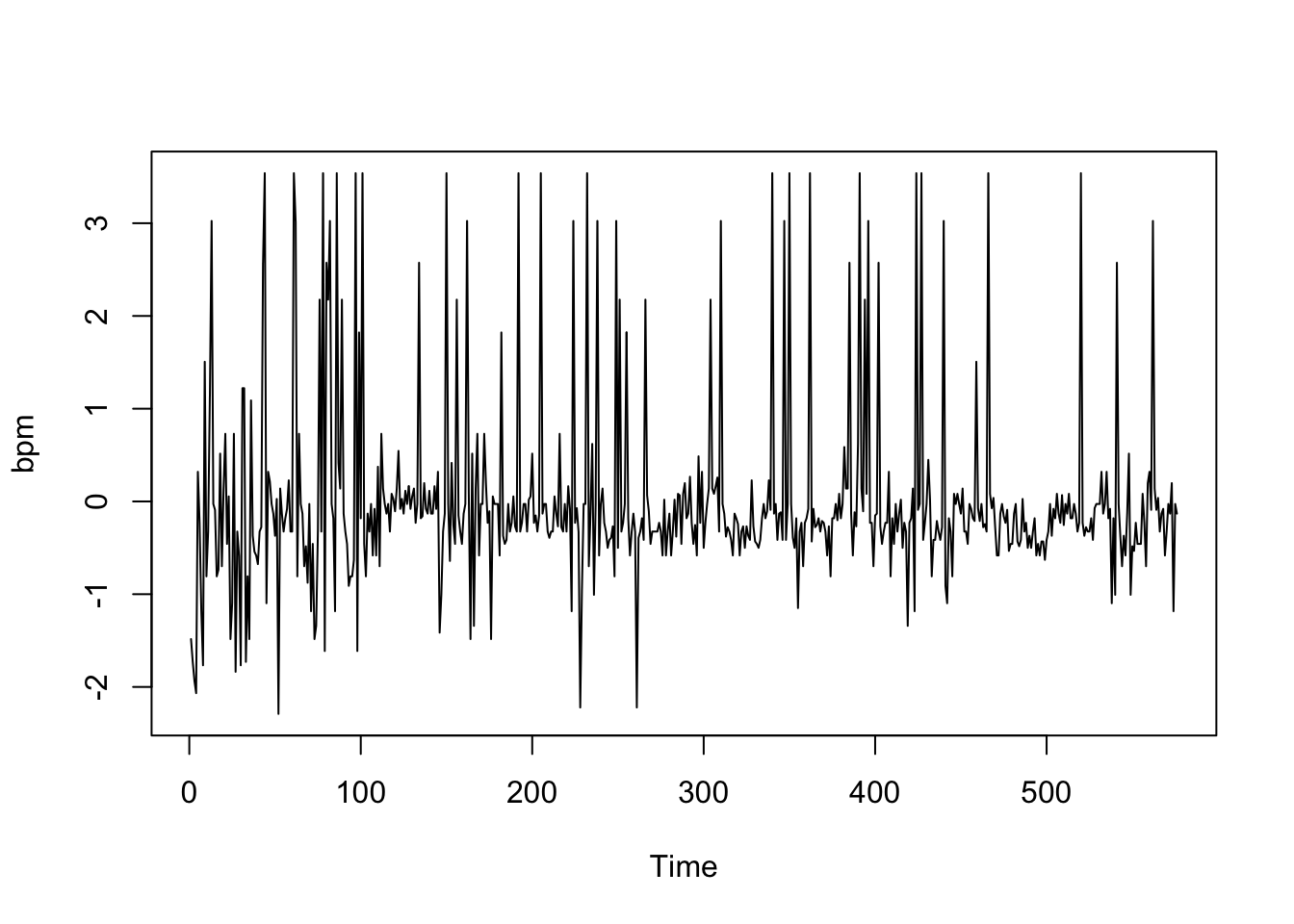


ccfvalues1<-ccf(pupil,bpm)

ccfvalues1

##

## Autocorrelations of series 'X', by lag

##

## -24 -23 -22 -21 -20 -19 -18 -17 -16 -15 -14

## -0.035 -0.079 -0.070 -0.057 -0.033 -0.066 -0.056 -0.020 0.007 0.030 0.021

## -13 -12 -11 -10 -9 -8 -7 -6 -5 -4 -3

## 0.050 0.022 0.024 0.057 0.040 0.016 -0.023 -0.015 -0.013 -0.018 -0.057

## -2 -1 0 1 2 3 4 5 6 7 8

## -0.020 -0.041 -0.056 -0.030 0.022 0.055 0.000 0.041 0.012 0.028 0.006

## 9 10 11 12 13 14 15 16 17 18 19

## 0.024 0.057 0.033 0.064 0.096 0.062 0.019 0.012 -0.005 0.009 0.012

## 20 21 22 23 24

## 0.028 0.011 0.052 -0.005 0.071

plot(ccfvalues1)

*#lag2.plot (pupil, bpm, 20)*

ccfvalues2<-ccf(pupil,temp)

ccfvalues2

##

## Autocorrelations of series 'X', by lag

##

## -24 -23 -22 -21 -20 -19 -18 -17 -16 -15 -14 -13 -12

## 0.137 0.137 0.140 0.142 0.148 0.148 0.151 0.152 0.154 0.153 0.152 0.151 0.154

## -11 -10 -9 -8 -7 -6 -5 -4 -3 -2 -1 0 1

## 0.157 0.166 0.168 0.171 0.169 0.170 0.169 0.164 0.154 0.143 0.137 0.136 0.137

## 2 3 4 5 6 7 8 9 10 11 12 13 14

## 0.128 0.125 0.129 0.137 0.139 0.139 0.133 0.125 0.129 0.131 0.137 0.129 0.115

## 15 16 17 18 19 20 21 22 23 24

## 0.106 0.091 0.080 0.072 0.063 0.050 0.045 0.042 0.037 0.030

plot(ccfvalues2)

*#lag2.plot (pupil,temp, 10)*

alldata=ts.intersect(pupil,pupillag1=lag(pupil,-1), pupillag2=lag(pupil,-2),

pupillag3=lag(pupil,-3),

templag1 = lag(temp,-1),

templag2 = lag(temp,-2),

templag3 = lag(temp,-3),

templag4 = lag(temp,-4),

templag5 = lag(temp,-5),

templag6=lag(temp,-6),

templag7=lag(temp,-7),

templag8=lag(temp,-8),

templag9=lag(temp,-9),

templag10=lag(temp,-10),

templag11=lag(temp,-11),

templag12=lag(temp,-12),

templag13=lag(temp,-13),

templag14=lag(temp,-14),

templag15=lag(temp,-15),

templag16=lag(temp,-16),

templag17=lag(temp,-17),

templag18=lag(temp,-18),

templag19=lag(temp,-19),

templag20=lag(temp,-20),

bpmlag1 = lag(bpm,-1),

bpmlag2 = lag(bpm,-2),

bpmlag3 = lag(bpm,-3),

bpmlag4 = lag(bpm,-4),

bpmlag5 = lag(bpm,-5),

bpmlag6=lag(bpm,-6),

bpmlag7=lag(bpm,-7),

bpmlag8=lag(bpm,-8),

bpmlag9=lag(bpm,-9),

bpmlag10=lag(bpm,-10),

bpmlag11=lag(bpm,-11),

bpmlag12=lag(bpm,-12),

bpmlag13=lag(bpm,-13),

bpmlag14=lag(bpm,-14),

bpmlag15=lag(bpm,-15),

bpmlag16=lag(bpm,-16),

bpmlag17=lag(bpm,-17),

bpmlag18=lag(bpm,-18),

bpmlag19=lag(bpm,-19),

bpmlag20=lag(bpm,-20)

)

regmodel = lm(pupil~pupillag1+pupillag2+templag8+templag9+bpmlag3, data = alldata)

summary(regmodel)

##

## Call:

## lm(formula = pupil ~ pupillag1 + pupillag2 + templag8 + templag9 +

## bpmlag3, data = alldata)

##

## Residuals:

## Min 1Q Median 3Q Max

## -2.97434 -0.49181 0.01207 0.46422 2.51645

##

## Coefficients:

## Estimate Std. Error t value Pr(>|t|)

## (Intercept) 0.004206 0.032530 0.129 0.897171

## pupillag1 0.538914 0.041946 12.848 < 2e-16 ***

## pupillag2 0.148211 0.042049 3.525 0.000459 ***

## templag8 0.843729 0.388758 2.170 0.030410 *

## templag9 -0.819055 0.383759 -2.134 0.033260 *

## bpmlag3 0.046323 0.032562 1.423 0.155421

## ---

## Signif. codes: 0 '***' 0.001 '**' 0.01 '*' 0.05 '.' 0.1 ' ' 1

##

## Residual standard error: 0.7553 on 550 degrees of freedom

## Multiple R-squared: 0.425, Adjusted R-squared: 0.4198

## F-statistic: 81.31 on 5 and 550 DF, p-value: < 2.2e-16

acf2(residuals(regmodel))

## [,1] [,2] [,3] [,4] [,5] [,6] [,7] [,8] [,9] [,10] [,11] [,12] [,13]

## ACF 0 -0.05 -0.04 0.03 0.1 0 0.04 0.00 -0.03 0.11 0.09 0.03 0.03

## PACF 0 -0.05 -0.04 0.03 0.1 0 0.06 0.01 -0.04 0.10 0.08 0.03 0.05

## [,14] [,15] [,16] [,17] [,18] [,19] [,20] [,21] [,22] [,23] [,24] [,25]

## ACF 0.01 -0.03 0.04 0.03 0.08 -0.03 0.00 0.03 0.05 0.03 -0.02 0.03

## PACF 0.02 -0.05 0.03 0.01 0.06 -0.02 0.01 0.01 0.04 0.01 -0.03 0.03

## [,26] [,27] [,28] [,29] [,30] [,31] [,32] [,33] [,34]

## ACF 0.00 -0.04 -0.02 0.00 0.04 0.03 0.03 -0.02 -0.07

## PACF -0.01 -0.06 -0.04 -0.01 0.03 0.04 0.03 -0.02 -0.07
